# Impact of the Three Gorges project on ecological environment changes and snail distribution in Dongting Lake area

**DOI:** 10.1371/journal.pntd.0005661

**Published:** 2017-07-06

**Authors:** Feiyue Li, Shujuan Ma, Yiyi Li, Hongzhuan Tan, Xunya Hou, Guanghui Ren, Kaiping Cai

**Affiliations:** 1 Department of Epidemiology and Health Statistics, Xiangya School of Public Health, Central South University, Changsha, China; 2 Department of Prevention and Control, Hunan Institute of Schistosomiasis Control, Yueyang, China; 3 Department of Science and Education, Hunan Institute of Schistosomiasis Control, Yueyang, China; Federal University of Agriculture, NIGERIA

## Abstract

**Background:**

The Three Gorges Dam (TGD) is a remarkable, far-reaching project in China. This study was conducted to assess the impact of TGD on changes in the ecological environment, snail distribution and schistosomiasis transmission in Dongting Lake area.

**Methods:**

Hydrological data were collected from 12 monitoring sites in Hunan section of Yangtze River before and after TGD was established. Data on snail distribution and human schistosomiasis infection were also collected. Correlation analyses were performed to detect the significance of snail distribution to changes in ecological environmental factors and human schistosomiasis infection.

**Findings:**

A series of ecological environmental factors have changed in Dongting Lake area following the operation of TGD. Volume of annual runoff discharged into Dongting Lake declined by 20.85%. Annual sediment volume discharged into the lake and the mean lake sedimentation rate decreased by 73.9% and 32.2%, respectively. From 2003 to 2015, occurrence rate of frames with living snails and mean density of living snails decreased overall by 82.43% and 94.35%, respectively, with annual decrements being 13.49% and 21.29%. Moreover, human infection rate of schistosomiasis had decreased from 3.38% in 2003 to 0.44% in 2015, with a reduction of 86.98%. Correlation analyses showed that mean density of living snails was significantly associated with water level (r = 0.588, *p*<0.001), as well as the mean elevation range of the bottomland (r = 0.374, *p* = 0.025) and infection rate of schistosomiasis (r = 0.865, *p*<0.001).

**Conclusion:**

Ecological environmental changes caused by the TGD were associated with distribution of snails, and might further affect the transmission and prevalence of schistosomiasis. Risk of schistosomiasis transmission still exists in Dongting Lake area and long-term monitoring is required.

## Author summary

Three Gorges Dam (TGD) is a tremendous hydrological project in China, it affects the ecological environment and influences the survival of animals downstream. The Dongting Lake is the first large lake that connects Yangtze River after it flows out of TGD, meanwhile, it accounts for 48% of the total snail habitation in China. Hydrological data from 12 monitoring sites, combined with data on snail distribution and human schistosomiasis infection, were used to assess the impact of TGD on ecological environment changes, snail distribution and schistosomiasis transmission in Dongting Lake area. Based on our analyses, following the establishment of TGD until 2015, the mean density of living snails in Dongting Lake area was under 5 snails/frame, and human infection rate of schistosomiasis declined to below 0.5%. As the impact of TGD on snail distribution and schistosomiasis prevalence in Dongting Lake area is much more complex in the real world, long-term monitoring and in-depth studies are still required.

## Introduction

The Three Gorges Dam (TGD) is located at the upper reaches of the Yangtze River, and it is a remarkable, comprehensive hydropower project [1]. Sediment discharge and water impoundment at the dam started in 2003. Following the cofferdam power generation period (2003–2005, water level 135 m), initial operation period (2006–2007, water level 156 m), and trial impoundment period (2008–2010, water level 185 m), the TGD became fully operational in 2010 [2]. Dongting Lake is the first large lake that connects Yangtze River after it outflows TGD, and it is the most important flood control lake in the middle reaches of Yangtze River [3]. Moreover, the Dongting Lake area is one of the most severe schistosomiasis endemic areas in China, as it has vast marshlands which are habitations for *Oncomelania hupensis* populations, the host of *Schistosoma japonicum (S*.*japonicum)* [4]. Although remarkable disease control efforts have been constantly conducted in the past decades, schistosomiasis remains endemic in five provinces (Hunan, Jiangsu, Hubei, Anhui and Jiangxi) along the Yangtze River [5, 6]. Currently, 1.759×10^9^ m^2^ of Dongting Lake area is infested with snails, which accounts for 48% of the total snail habitation in China [7]. Risk of schistosomiasis transmission still exists in this area [2, 8–10].

Large-scale hydro-projects not only affect the natural environment, but may also alter regional climate, and further lead to changes in the epidemic distribution of some infectious and endemic diseases [11, 12]. Previous cases have shown that emergence or re-emergence of schistosomiasis were often caused by newly-built hydro-projects in endemic areas, such as Aswan Dam in Egypt, Tigay Dam in Ethiopia, Gezira-Managil Dam in Sudan, Manantali Dam in Mali and Danling Dam in China [11–13]. Hence, the potential influence of TGD on the transmission of schistosomiasis in downstream Yangtze River basin aroused heated discussion worldwide [2, 12, 14, 15]. During the past decades, lots of studies have been conducted to forecast or assess the influence of TGD on the distribution of snails and transmission of schistosomiasis, but the conclusions were inconsistent [2, 4, 12, 14, 16, 17]. Therefore, this study was conducted to analyze the impact of TGD on the ecological environment, snail distribution and schistosomiasis transmission, respectively. And it aims to provide evidence for the control and elimination of schistosomiasis.

## Methods

### Study sites

**Fig 1:**
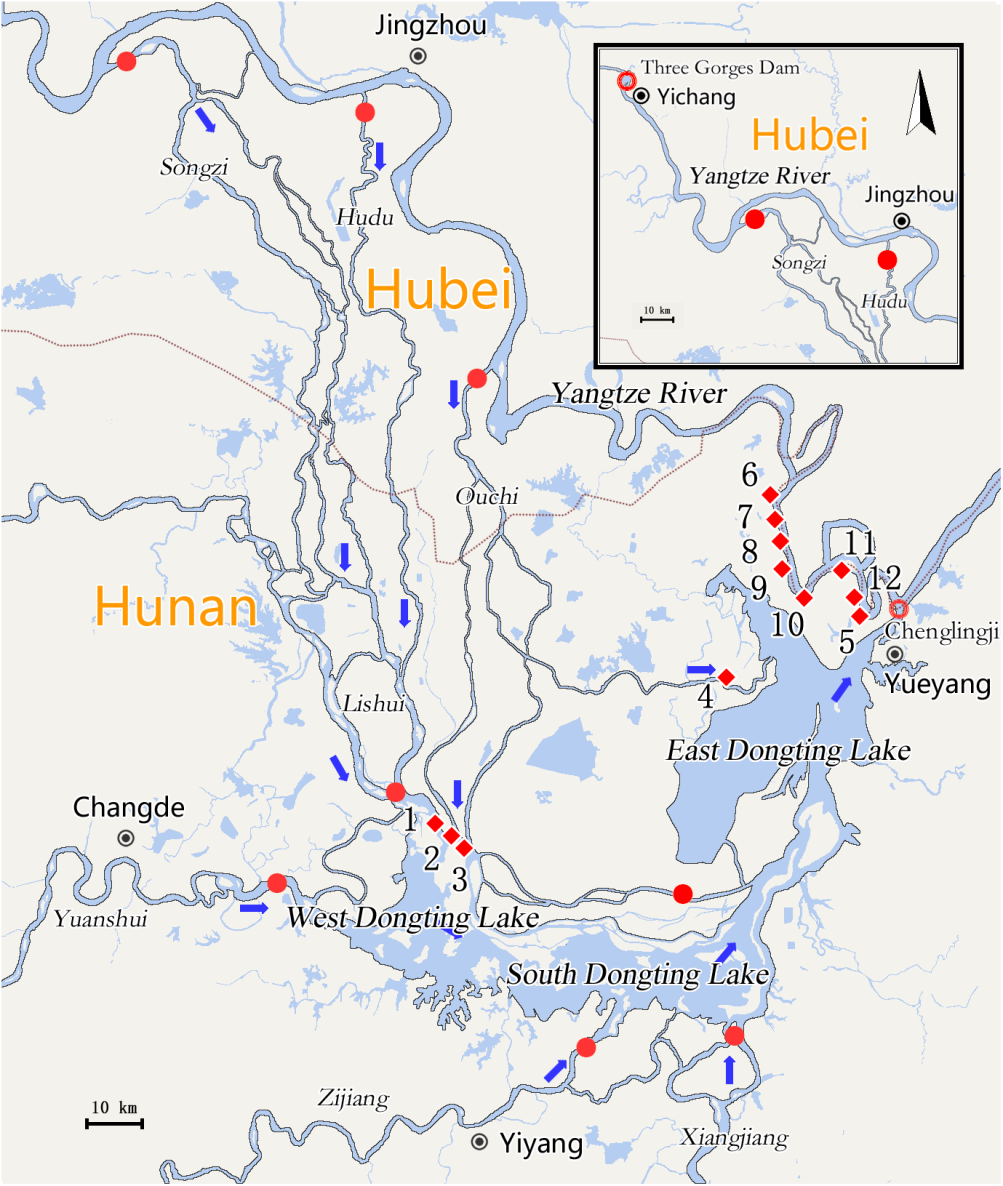
Maps of Three Gorges Dam, Yangtze River and Dongting Lake. Blue arrows represented flow direction of rivers or lakes; Red dots represented diversion channels of Yangtze River, and four tributaries (Xiang, Zi, Yuan and Li rivers) into Dongting Lake; Red circles represented outlets of Dongting Lake into the Yangtze River; Red diamond and surrounding Arabic numeral represented locations of each monitoring site: 1→Songzi estuary, 2→Ouchi (middle branch) estuary, 3→Ouchi (Tuojiang river) estuary, 4→Ouchi (east branch) estuary, 5→Dongting Lake outlet, 6→Water gate of Hongshuigang, 7→Water gate of Tanzikeng, 8→Water gate of Sizhiqu, 9→Water gate of Liuzhiqu, 10→Water gate of Jingjiangmen, 11→Water gate of Bei, 12→Water gate of Aiwei.

This study was conducted in the Dongting Lake region. The lake is located in the northern part of Hunan province and it encompasses a water surface area of 2681 km^2^. In the southeast, the lake is fed by four tributaries (Xiang, Zi, Yuan, and Li) in Hunan province. In the north, it collects water from Yangtze River via three outlets (Songzi, Taiping, and Ouchi), and returns outflow into Yangtze River at Chenglingji in Yueyang City [18]. The marshland where snails are distributed were selected as the study sites. A total of 12 monitoring sites, were established for this study. These sites were located on the estuaries of Yangtze River (Fig 1), including bottomlands outside the embankment and polder ditches inside the protective embankment of Yangtze River (n = 7, including water gates of Hongshuigang, Tanzikeng, Sizhiqu, Liuzhiqu, Jingjiangmen, Bei and Aiwei), as well as bottomlands on diversion channels (n = 5, including Songzi estuary, middle Ouchi branch estuary, Ouchi Tuojiang river estuary, east Ouchi branch estuary and Dongting Lake outlet). Global Positioning System information of each monitoring site is presented in S1 Table.

### Data collection

Hydrological information was provided by hydraulic department of the government to evaluate the impact of TGD construction and impoundment during the preceding years. Information pertaining to Dongting Lake regarding volumes of runoff and sediment, water level, and topsoil moisture level were also collected. Marshland moisture was recorded annually in March and April from 2003 to 2015, concurrently with the snail investigation. Nine points were selected for each monitoring site, including four in the corners and four at the midpoints of each line of the frame, as well as one in the middle. A field TDR 300 soil moisture meter (Spectrum Tech, USA) was used to measure topsoil moisture of the marshland.

Snail data including the total areas surveyed, number of frames, number of living snails and number of infected snails were collected annually in March and April from 2003 to 2015. For bottomlands outside the embankments, the snail survey was conducted using a traditional random equidistant frame survey method (0.11 m^2^-sized frames, 20 meters apart between frames) [19]. While for areas around canal branches, sub-branches, and ditches inside the embankments, the frames were set at 0.5 m × 10 m. Records were also taken about the name of marshland, administrative government, water body, as well as the elevation range of marshland where the survey sites are located. The appearance of cercariae in the crushed snail samples when observed under the microscope indicate that the snail has infection and they are defined as infected snails.

Data on human schistosomiasis infection in Hunan province were extracted from the statistical annual report of Hunan Institute of Schistosomiasis Control. Data were collected through county (city, district, farm) -based field surveys in epidemic areas. The study scope were inhabitants aged 6–65 years old living in 41 counties (cities, districts, farms) of Hunan province. These inhabitants were checked every 1–3 years from 2003 to 2015, according to different categories of epidemic regions.

### Statistical analysis

A database was established using Microsoft Excel. Changes in snail distribution, runoff volume, sediments, water level in Dongting Lake area, and resident infection rate of schistosomiasis in Hunan province, were visually described with statistical tables and line graphs. Changes in trend of occurrence rate of frames with living snail was detected using Chi-square trend test. Correlation analyses were performed to detect whether snail distribution was relevant to changes in ecological environmental factors and human schistosomiasis infection. All *p* values were two-sided, with a significant level of *p* ≤ 0.05. Statistical analyses were performed using SPSS version 23.0.

## Results

### Hydraulic changes in Dongting Lake area

**Fig 2:**
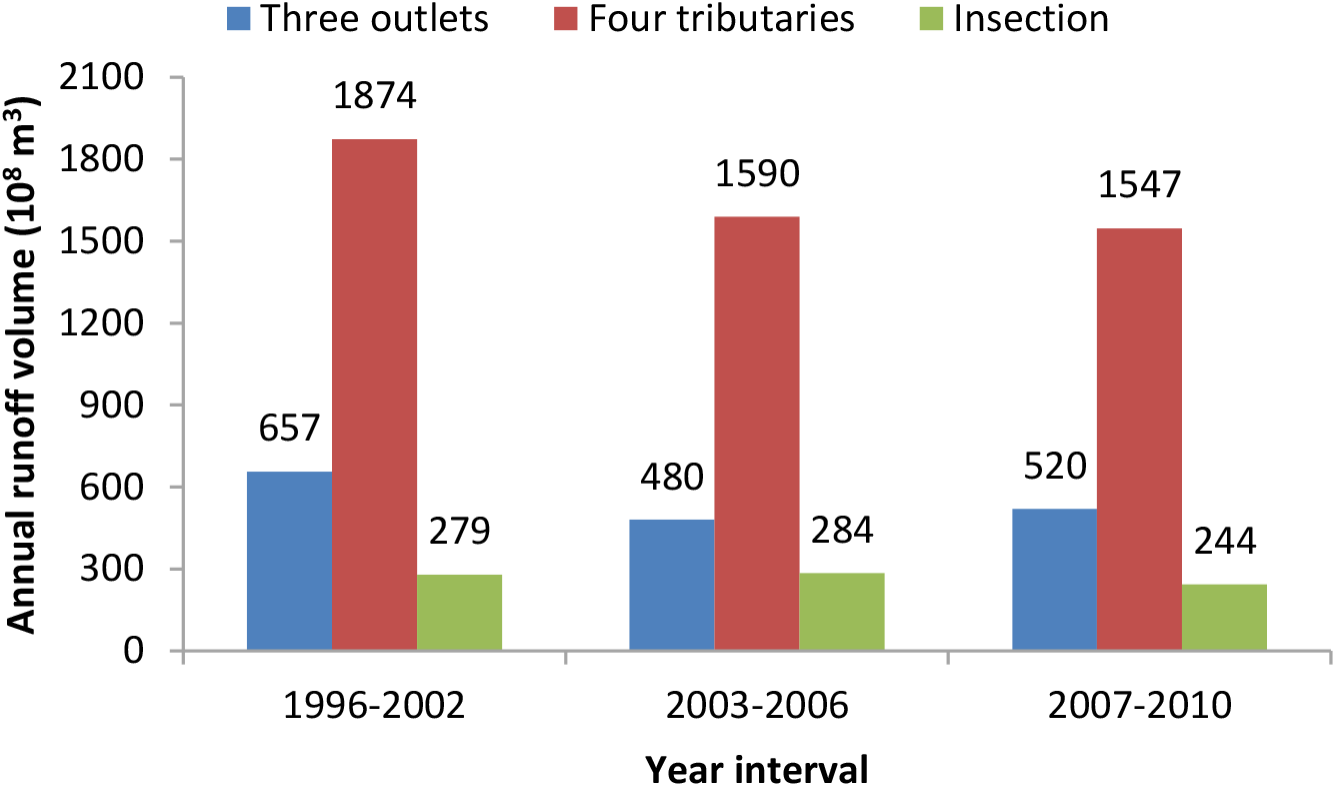
Annual runoff volume (10^8^ m^3^) of Dongting Lake before and after the operation of the Three Gorges Dam. Three outlets represented Songzi, Taipin, and Ouchi. Four tributaries represented Xiang, Zi, Yuan and Li rivers.

The mean annual runoff volume that drained into Dongting lake from three outlets of Yangtze River was 657 (10^8^ m^3^) from 1996 to 2002. After the construction of TGD in 2003, water discharged into the lake decreased gradually, and the mean annual runoff volume decreased to 520 (10^8^ m^3^) in 2007–2010, equal to a reduction of 20.85% compared to that before the impoundment. Duration of dry-up days in eastern branches of Songzi and Ouchi rivers, and in western branches of Hudu and Ouchi rivers had increased from 92, 117, 242 and 117 in 1996–2002 to 199, 190, 257 and 152 in 2003–2008, respectively. Mean annual runoff volume of four tributaries in Hunan Province and Dongting Lake declined from 1874 (10^8^ m^3^) and 279 (10^8^ m^3^) in 1996–2002, to 1574 (10^8^ m^3^) and 244 (10^8^ m^3^) in 2007–2010, with reduction proportion of 17.45% and 14.76%, respectively (Fig 2).

**Fig 3:**
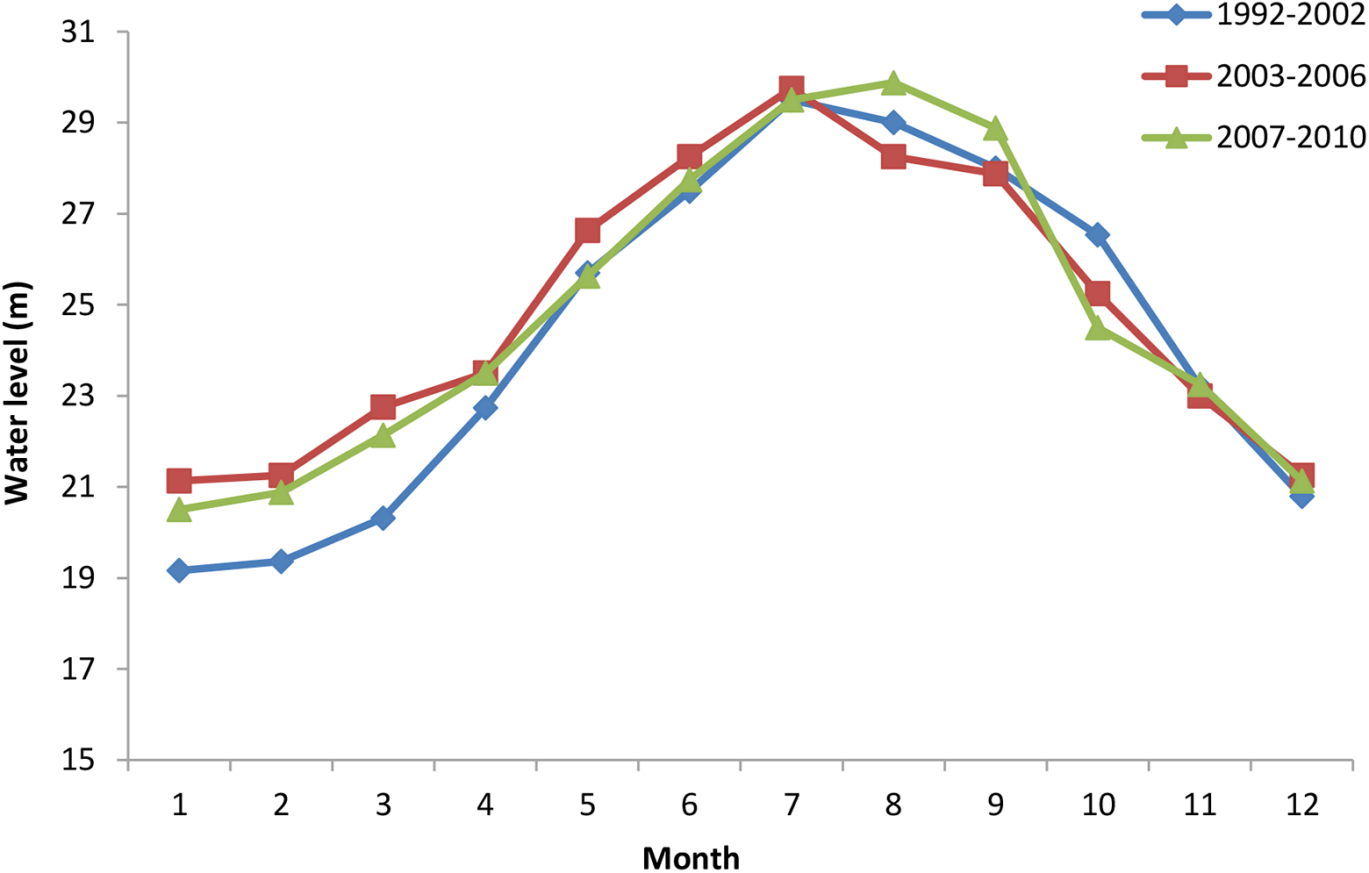
Mean monthly water level at different year intervals at Chenglingji station of Dongting Lake.

During the impoundment period of TGD from mid-September to the end of October, volume of water drained from the mainstream of Yangtze River decreased dramatically, this led to rapid reduction of Dongting Lake water level, then resulted into an advanced dry season in Yangtze River and Dongting Lake. However, water level in Dongting Lake during the dry season (from December to April) was found higher than before TGD (1992–2002). Thus, by comparing data collected after the establishment of TGD (2007–2010) with that before it, the mean monthly water level at Chenglingji (outlet of Dongting Lake) was reduced by 2.04m in October, but increased by 0.34m, 1.34m, 1.52m, 1.82m, and 0.76m in December, January, February, March and April, respectively (Fig 3).

In the pre-TGD period (1996–2002), the mean annual sediment discharge volume of the lake basin was 85.43 million tons, and the sedimentation rate was 73.7%. Following the TGD project (2003–2010), these metrics changed to 22.28 million tons and 41.5%, respectively. Compared with the data collected before TGD, annual sediment discharge declined by 73.9%, 90.5% of which was derived from the three outlets and 9.5% was from the four tributaries. Moreover, the sedimentation rate of the lake basin declined by 32.2%. Table 1 depicts sediment discharge volume and lake basin sedimentation rate in Dongting Lake before and after TGD.

**Table 1:** Annual sediment volume of Dongting Lake before and after the establishment of Three Gorges Dam.

Stages of Three Gorges Dam	Statistical years and detailed stages	Mean annual sediments discharged into the lake (10^4^ tons)			Mean annual sediments discharged out of the lake (10^4^ tons)	Mean annual sediments in the basin (10^4^ tons)	Mean annual sedimentation rate (%)
		Three outlets	Four tributaries	Total	Chenglingji	Dongting Lake	Dongting Lake
Before operation	1996–2002	6959	1584	8543	2251	6292	73.7
After operation	Come into operation (2003–2005)	1967	1039	2228	1590	1416	41.5
	Early stage of operation (2006–2007)	746	921		1000	667	
	Trial period of impoundment (2008–2010)	851	974		1219	606	

Note: The data was based on hydrological data recorded at the hydrological stations at three outlets (Songzi, Taiping, and Ouchi) of Yangtze River in Jingjiang, four tributaries (Xiang, Zi, Yuan and Li rivers) in Hunan, and Hukou of Dongting Lake.

### Environmental changes in the bottomlands of Dongting Lake

The highest and lowest elevation ranges integrally decreased following the establishment of TGD, respectively from 30.40–34.90 m and 23.00–30.40 m to 29.10–34.50 m and 21.00–30.20 m. Meanwhile, the mean elevation range increased slightly after the operation of TGD, from 26.95–31.50 m to 28.20–32.35 m, but the change was not statistically significant. Moreover, mean topsoil moisture of bottomland measured from the survey sites was 46.4% (range: 35.50–50.10%) prior to the establishment of TGD, and slightly increased to 47.30% (41.40–53.40%) after the establishment of TGD, but no statistical significance was found.

### Changes in snail distribution

**Fig 4:**
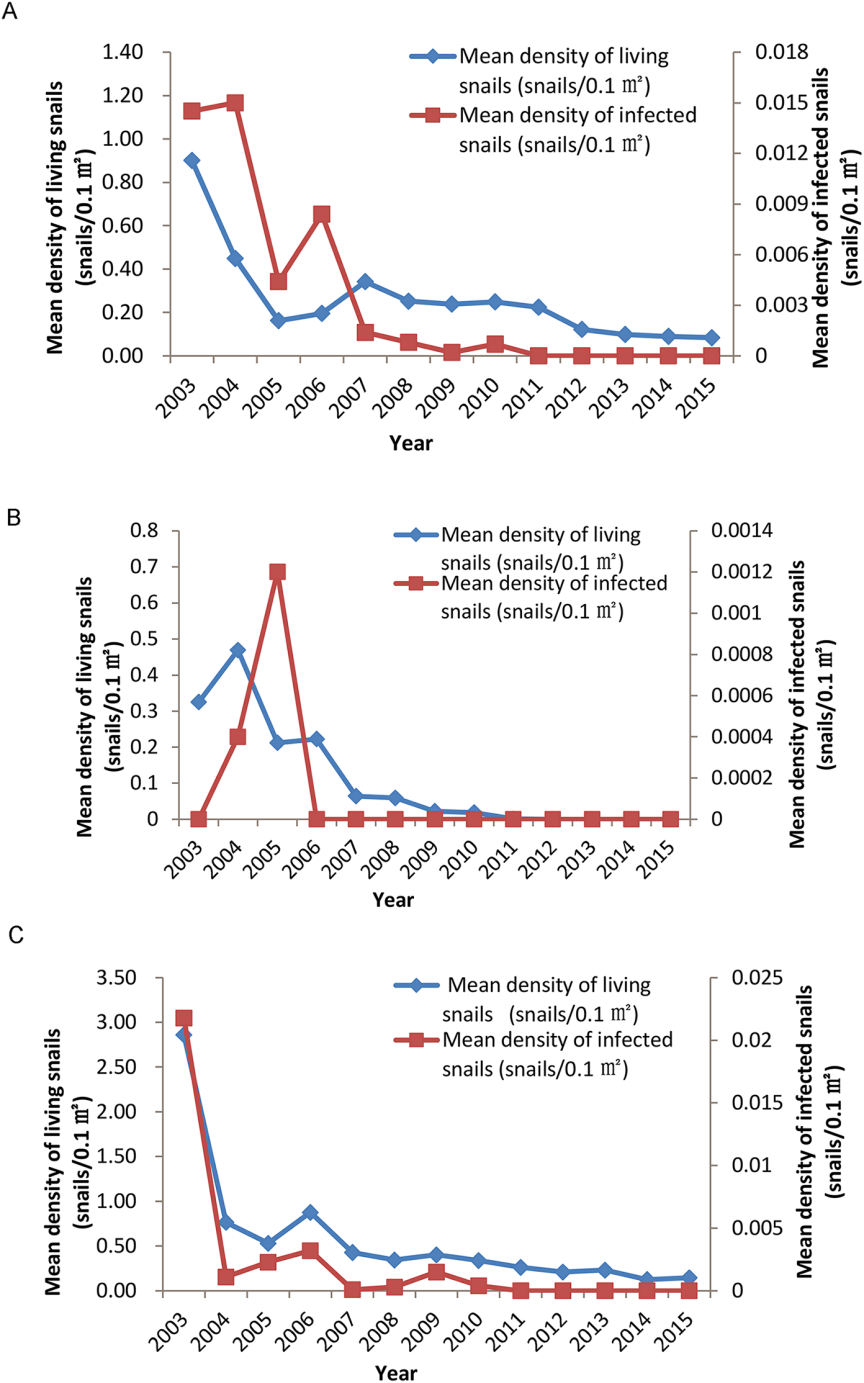
Distribution of snails in bottomlands outside the embankment of Yangtze River in Hunan section (A), in polder ditches inside the protective embankment of Yangtze River (B), and in bottomlands on diversion channels of Yangtze River (C) from 2003 to 2015.

Snail distribution result in the 12 monitoring sites is presented in Table 2. A total of 12 monitoring sites were surveyed at Dongting Lake, the occurrence rate of frames with living snails (*Oncomelania hupensis*) declined from 26.87% in 2003 (before the TGD project) to 4.72% in 2015 (after the TGD project), with reduction rate being 82.43% and annual decrement being 13.49%. Detailed data showed that occurrence rates of frames with living snails in bottomlands on diversion channels were relatively high (9.18–48.39%), and the rates of that in polder ditches inside the protective embankment of Yangtze River were the lowest (0–11.65%). Following the establishment of TGD, the occurrence rate of frames with living snails decreased gradually with significant linear tendency (χ^2^ = 7053.05, p<0.001). Similarly, the mean density of living snails declined from 1.2140 snail/frame in 2003 to 0.0686 snail/frame in 2015, indicating a reduction rate of 94.35% and annual decrements of 21.29%. Until 2012, the mean density of living snails had dropped to zero in the monitoring polder ditches inside the protective embankment of seven sites (Fig 4A). Meanwhile, the mean density of living snails in bottomlands outside the embankment of the same sites and of bottomlands on diversion channels of the other five sites had also dropped to a relatively low level (Fig 4B and 4C).

**Table 2:** Snail distribution in the monitoring sites of the Yangtze River in Hunan section from 2003 to 2015.

Year	Bottomlands outside the embankment of Yangtze River (7 sites)						Polder ditches inside the protective embankment of Yangtze River (7 sites)						Bottomlands on diversion channels (5 sites)						Overall (12 sites)					
	A	B	C	D	E	F	A	B	C	D	E	F	A	B	C	D	E	F	A	B	C	D	E	F
2003	308.6	7710	26.36	0.9000	1.31	0.0145	18.3	3894	8.17	0.3246	0	0	189.4	3571	48.39	2.8617	1.82	0.0218	516.3	15175	26.87	1.2140	1.09	0.0125
2004	869.0	9768	21.46	0.4486	1.48	0.0150	23.7	4730	9.43	0.4693	0.04	0.0004	411.9	2720	33.79	0.7643	0.07	0.0011	1304.6	17218	20.10	0.5042	0.87	0.0088
2005	740.6	18542	8.74	0.1619	0.44	0.0044	21.7	4928	10.78	0.2121	0.12	0.0012	389.4	8809	19.13	0.5293	0.23	0.0023	1151.7	32279	11.88	0.2698	0.33	0.0033
2006	742.6	18604	9.61	0.1958	0.78	0.0084	19.7	4360	11.65	0.2222	0	0	379.4	8749	31.43	0.8755	0.16	0.0032	1141.6	31713	15.91	0.3869	0.50	0.0058
2007	640.0	16095	14.10	0.3420	0.14	0.0014	20.9	5462	5.58	0.0639	0	0	371.9	8114	21.00	0.4275	0.01	0.0001	1032.8	29671	14.42	0.3142	0.08	0.0008
2008	679.0	17100	10.17	0.2522	0.06	0.0008	23.1	7322	5.41	0.0593	0	0	311.9	6398	17.36	0.3448	0.03	0.0003	1014.0	30820	10.53	0.2256	0.04	0.0005
2009	769.0	19380	10.61	0.2389	0.01	0.0002	22.6	7321	1.58	0.0228	0	0	367.4	6673	19.65	0.4009	0.12	0.0015	1159.0	33374	10.44	0.2239	0.03	0.0004
2010	909.0	22844	12.40	0.2481	0.07	0.0007	23.1	7497	1.28	0.0191	0	0	387.4	8494	16.11	0.3397	0.04	0.0004	1319.5	38835	11.06	0.2239	0.05	0.0005
2011	909.0	22802	10.98	0.2237	0.00	0	19.9	7014	0.09	0.0010	0	0	321.5	8275	13.00	0.2620	0	0	1250.4	38091	9.41	0.1910	0	0
2012	339.8	7570	8.35	0.1219	0	0	17.9	13280	0	0	0	0	156.0	4091	12.71	0.2095	0	0	513.7	24941	4.62	0.0714	0	0
2013	310.0	7750	7.45	0.0990	0	0	24.9	6300	0	0	0	0	156.0	4031	13.79	0.2322	0	0	490.9	18081	6.27	0.0942	0	0
2014	310.0	7750	6.72	0.0901	0	0	46.0	6300	0	0	0	0	156.0	4031	9.48	0.1253	0	0	512.0	18081	4.99	0.0665	0	0
2015	310.0	7750	6.25	0.0841	0	0	46.0	6300	0	0	0	0	156.0	4031	9.18	0.1459	0	0	512.0	18081	4.72	0.0686	0	0
G	-	-	11.30	17.92	-	-	-	-	-	-	-	-	-	-	12.94	21.97	-	-	-	-	13.49	21.29	-	-
H	-	-	76.29	90.66	100	100	-	-	100	100	100	100	-	-	81.03	94.90	100	100	-	-	82.43	94.35	100	100

Note: A, monitored area (hm^2^); B, number of frames (0.11 m^2^); C, occurrence rate of frames with living snails (%); D, mean density of living snails (snails/ 0.11m^2^); E, occurrence rate of frames with infected snails (%); F, mean density of infected snails (snails/ 0.11m^2^); G, yearly reduction rate (%); H, total reduction (%).

All the sampled living snails were screened to identify infected ones. A total of 190 infected snails were identified from 166 frames with living snails in all the sites monitored in 2003, of which, 101 frames were from bottomlands outside the embankment of seven sites. Following the establishment of TGD, the occurrence rate of frames with infected snails decreased from 1.09% in 2003 to 0.05% in 2010, with a significant linear decreasing tendency (χ^2^ = 996.81, p<0.001). The corresponding mean density of infected snails has been decreasing and finally reached a low level (0.0008 snails/ 0.11m^2^) in 2007, then the value became zero in 2011 (Fig 4A, 4B and 4C).

### Human schistosomiasis infection in Dongting Lake area

Data about human schistosomiasis infection in Dongting Lake area is shown in Table 3. From 2003 to 2015, the human schistosomiasis epidemic had been well controlled, a total of 256 villages had been out of the human schistosomiasis infection throughout the Hunan province. More than 5×10^5^ persons were randomly selected to check for schistosomiasis infection each year. The infection rate of schistosomiasis had steady decreased from 3.38% in 2003 to 0.44% in 2015, with a reduction rate of 86.98%. Based on the stable population, the decreased infection rate of schistosomiasis was due to the decline in the number of schistosomiasis patients, the reduction rate for the latter was 86.41%.

**Table 3:** Human schistosomiasis infection in Hunan province from 2003 to 2015.

Year	Number of epidemic villages	Number of detected persons	Infection rate of schistosomiasis (%)	Total population of epidemic villages (million)	Number of schistosomiasis patients
2003	3987	724020	3.38	6.1005	205461
2004	3942	504836	3.30	6.2167	211615
2005	3875	563442	3.04	6.3251	211940
2006	3842	612556	2.78	6.4334	178956
2007	3867	668361	1.98	6.4330	127614
2008	3698	656548	1.46	6.3517	92932
2009	3698	609247	1.49	6.3680	94811
2010	3701	792355	1.37	6.4632	88229
2011	3706	799674	1.22	6.4246	78480
2012	3702	928210	0.97	6.4402	62479
2013	3704	1081364	0.65	6.4403	41877
2014	3703	902136	0.50	6.3911	31669
2015	3731	867099	0.44	6.3458	27930

### Correlation analyses

Correlation analysis was performed between three monitored ecological environmental factors (water level, mean elevation range, and topsoil moisture) and occurrence rate of frames with living snails, as well as mean density of living snails (Table 4). Results suggested that the monitored water level was significantly associated with occurrence rate of frames with living snails (r = 0.509, *p* = 0.002) and the mean density of living snails (r = 0.588, *p*<0.001). Moreover, mean elevation range of the bottomland was found to be significantly associated with mean density of living snails (r = 0.374, *p* = 0.025). In addition to these, no other statistical association was found, snail distribution was not significantly associated with the topsoil moisture (Table 4). However, index on snail distribution were found to be significantly associated with infection rate of schistosomiasis and number of schistosomiasis patients, with the correlation coefficients ranging from 0.718 to 0.865 (Table 4).

**Table 4:** Correlation between snail distribution and changes in ecological environmental factors, as well as human schistosomiasis infection in Dongting Lake area.

Snail distribution	Ecological environmental factors						Human schistosomiasis infection			
	Monitored water level of the bottomlands		Mean elevation range of the bottomlands		Topsoil moisture		Infection rate of schistosomiasis		Number of schistosomiasis patients	
	r	*p* value	r	*p* value	r	*p* value	r	*p* value	r	*p* value
Occurrence rate of frames with living snails	0.509	0.002	0.274	0.105	0.135	0.433	0.795	<0.001	0.718	0.001
Mean density of living snails	0.588	<0.001	0.374	0.025	0.110	0.524	0.865	<0.001	0.787	<0.001

## Discussion

Based on the collected hydrological data and fixed-point monitoring data, our results confirmed that the TGD project had changed water and sand distribution downstream, impacting the ecological environment, snail distribution and schistosomiasis transmission in Dongting Lake area.

Due to the impoundment of TGD, from mid-September to the end of October, volume of water drained from Yangtze River mainstream decreased dramatically. Water level of Dongting Lake decreased rapidly while the volume of water flowing out of the lake increased, this resulted into a reduction in the total amount of lake water, which further exposed the bottomlands and also extended the dry season in this area. During the dry season (from December to April), water level in Dongting Lake was higher than that before the construction of TGD, as a result of the release of water from TGD reservoir, which was used to meet the needs of power generation and shipping. While during the wet season, the TGD could be used as a channel to reduce Dongting Lake water level thereby control flooding of the area. Moreover, the highest elevation and mean topsoil moisture of monitoring bottomlands in Dongting Lake slightly increased following the operation of TGD.

This study revealed a significant linear decrease in trend of snail populations in all the 12 sites monitored at Dongting Lake from 2003 to 2015. The occurrence rate of frames with living snails and mean density of living snails declined overall by 82.43% and 94.35%, respectively, with annual decrements being 13.49% and 21.29%, respectively. These results were in accordance with the longitudinal monitoring data of schistosomiasis in Hunan province [20]. Moreover, the reduction differed in different types of bottomlands. The polder ditches used to divert water from Yangtze River to irrigate farmlands inside the protective embankments of Dongting Lake had the largest amount of deduction, followed by bottomlands of Yangtze River in Hunan section, and estuaries of Yangtze River where river water entered the lake. At different stages of the TGD project, decline of snail density in bottomlands of Dongting Lake area varies. Overall decline was highest in impoundment period, snail density decreased drastically in polder ditches during impoundment period and early stage of project operation. During the normal operation period, snail distributions had the smallest changes both in bottomlands and polder ditches. This might be related to variable impacts of the impoundment of TGD on different areas of Dongting Lake, moreover, water level was constantly changing during the operation. Once the water in the Three Gorge Reservoir had reached a certain level, water level of the downstream areas, including Dongting Lake, would keep at a relatively constant level. Ecological environment of snails would then reach homoeostasis and the snail distribution would be equilibrated after dropping to a certain level.

Schistosomiasis is a natural environmental disease. Changes in natural environmental conditions have a significant impact on the growth, reproduction and spread of snails, and further impact the transmission of schistosomiasis [21]. At present, studies about environmental factors affecting snail distribution are mainly focused on hydrological characteristics, vegetation, water quality (especially water eutrophication), water temperature, PH value and so on [22–25]. Snail is an amphibian, living in moist, shaded and mixed wetland environment. Previous researches have showed that snail distribution was related to water level ranges, water coverage period, underground water level, and topsoil moisture [24, 26]. Correlation analyses in our study showed that monitored water level (r = 0.588, *p*<0.001) and mean elevation range of the bottomland (r = 0.374, *p* = 0.025) were significantly associated with mean density of living snails, which was in accordance with the report of Zheng and Wang *et al*. [26, 27].

The significant positive correlation between ecological environment changes and snail distribution might be interpreted as follows. Firstly, regulatory effect of TGD project reduced the variation between the highest and lowest water levels of Yangtze River and Dongting Lake. These may further change the microenvironment of snail habitats and curtail the infection to humans and animals [12, 15, 24]. Secondly, snail distribution in Dongting Lake area presents a feature of “two lines and three zones”. The “two lines” refers to the upper and lower lines containing living snails, while “three zones” refers to the lower sparse snail zone, dense snail zone, and the upper sparse snail zone. Along with the reduction in annual runoff volume into the lake, the highest water level of Dongting Lake area maintained under the lower sparse snail zone, which resulted in water shortage in snail habitation. As a result, the bottomland was no longer suitable for the growth of reeds, and further affected the survival rate of snails. Thirdly, as a result of the water discharged during the power generation of TGD, the lower sparse snail zone of the eastern Dongting Lake became submerged in spring (mainly in March and April). The advanced submerged snails and their eggs might likely affect their survival and further jeopardize the incubation of eggs, which eventually reduced the snail density. Moreover, some researchers believed that distribution of snails had an obvious seasonal variation, which was shown as two peaks in spring and the end of summer [28]. Water of TGD maintained at a relatively low level in summer and at a high level in winter, leading to a so-called “summer-land, winter-water” eco-hydrologic condition, which was opposite to the suitable environment for snails (“winter-land, summer-water”) [15].

*Oncomelania* is the only intermediate host of *Schistosoma japonicum*, and plays a critical role in the transmission process, its distribution will directly affect the prevalence of schistosomiasis [29]. Actually, our data had also confirmed the close relationship between snail distribution variation and the changes in human schistosomiasis infection, with the correlation coefficients ranging from 0.718 to 0.865. Moreover, our monitored data suggested that human infection rate of schistosomiasis and number of schistosomiasis patients in Dongting Lake area had greatly decreased following the establishment of TGD, till the year 2015, infection rate of schistosomiasis had dropped to 0.44%, which was under 1% as expected [4]. We believe that the remarkable disease control efforts conducted by the government, like extensive use of molluscicides, routine praziquantel treatment, health education programs, might have contributed significantly to the successful reductions in the intensity of living snails and human infection during the past decades [30, 31]. However, based on our analyses, we believe that a series of ecological environmental changes, including water level, bottomland soil moisture, microenvironment temperature, vegetation distribution and so forth, caused by TGD project can disturb distribution of snails, as well as the transmission and prevalence of schistosomiasis. Given that the impact of TGD on snail distribution and schistosomiasis prevalence in Dongting Lake area is much more complex, prolonged and in-depth studies are needed to address these issues for the effective control of snails in Dongting Lake area and eventually achieving the elimination of schistosomiasis.

## Supporting information

S1 Table Detailed information of each monitoring site. (DOCX)
