# Supplementary material for: Impact of the Three Gorges project on ecological environment changes and snail distribution in Dongting Lake area
Source: PLoS Negl Trop Dis. 2017 Jul 6;11(7):e0005661. doi: 10.1371/journal.pntd.0005661 (PMC5500280; doi:10.1371/journal.pntd.0005661)
Supplement: S1 Table — (DOCX) [file pntd.0005661.s001.docx]

| Supplementary Table 1. Detailed information of each monitoring site. | | | | | |
| --- | --- | --- | --- | --- | --- |
| Number | Monitoring site | Water system | Longitude (°E) | Latitude (°N) | Elevation range (m) |
| 1 | Songzi estuary | Songzi River | 112.2636 | 29.0884 | 28.5-34.5 |
| 2 | Ouchi (middle branch) estuary | Ouchi River | 112.3080 | 29.0702 | 28.5-32.4 |
| 3 | Ouchi (Tuojiang river) estuary | Ouchi River | 112.3253 | 29.0548 | 28.3-32.5 |
| 4 | Ouchi (east branch) estuary | Ouchi River | 112.8039 | 29.3257 | 21.0-30.4 |
| 5 | Dongting Lake outlet | Dongting Lake | 113.0764 | 29.4178 | 23.0-29.1 |
| 6 | Water gate of Hongshuigang | Yangtze River | 112.9016 | 29.6227 | 30.2-34.5 |
| 7 | Water gate of Tanzikeng | Yangtze River | 112.9085 | 29.5868 | 25.9-34.0 |
| 8 | Water gate of Sizhiqu | Yangtze River | 112.9158 | 29.5473 | 25.6-30.8 |
| 9 | Water gate of Liuzhiqu | Yangtze River | 112.9147 | 29.4988 | 30.0-31.0 |
| 10 | Water gate of Jingjiangmen | Yangtze River | 112.9556 | 29.4667 | 30.0-33.0 |
| 11 | Water gate of Bei | Yangtze River | 113.0258 | 29.5008 | 27.0-31.8 |
| 12 | Water gate of Aiwei | Yangtze River | 113.0518 | 29.4504 | 25.5-31.3 |
